# ﻿A new species of *Anthaxia* (Merocratus) Bílý, 1989 (Coleoptera, Buprestidae, Anthaxiini) from Vietnam

**DOI:** 10.3897/zookeys.1244.150712

**Published:** 2025-07-10

**Authors:** Mikuláš Plachetka

**Affiliations:** 1 Letňanská 330/15, 190 00, Prague, Czech Republic Unaffiliated Prague Czech Republic

**Keywords:** Anthaxiini, Buprestinae, identification key, jewel beetles, new record, Oriental region, taxonomy

## Abstract

A new species of genus Anthaxia (Merocratus) Bílý, 1989 from northern Vietnam is described and illustrated here: Anthaxia (Merocratus) rydzii**sp. nov.**, which belongs to the A. (M.) insulaecola Obenberger, 1944 species group. This medium-sized species (with respect to genus *Anthaxia*) varies in colour. Its elytra and pronotum could be green to violet, sometimes with a black tinge. It can be distinguished within the species group by the shape and structure of the pronotum, flat frons and, mainly, by missing a notch in the anal sternite which is a significant character and quite uncommon to subgenus Merocratus.

## ﻿Introduction

Subgenus Merocratus Bílý, 1989 was originally created for Anthaxia (M.) insulaecola Obenberger, 1944 and Anthaxia (M.) miranda Deyrolle, 1864. Two more species were described in the following years: A. (M.) bellisima Bílý, 1990 and A. (M.) jakli Bílý, 1996. The whole subgenus was revised by Bílý (1998), and in 2003 another species, A. (M.) karati Obořil & Bílý, 2003, was described. In 2019, Bílý and Plachetka established a species group Anthaxia (M.) insulaecola within the subgenus and classified all aforementioned species as members of this species group. Bílý and Plachetka described four more species of subgenus Merocratus in 2019, two of which belong to the newly established *A.insulaecola* species group. These species were: A. (M.) angustata Bílý, 2019 from north Vietnam and A. (M.) lucifera Plachetka, 2019 from Luzon, Philippines. In 2024, new *Anthaxia* specimens were collected in Vietnam, which turned out to belong to a new species described here: A. (M.) rydzii sp. nov. In 2025, another species belonging to the *A.insulaecola* species group was described: A. (M.) zhengyucheni Qi & Song, 2025, from Hainan (Qi et al. 2025).

In addition to the new species account, a new record and brief description of the female of A. (M.) angustata is also provided.

## ﻿Material and methods

A Canon 80D digital camera equipped with a Canon MP-E65mm f/2.8-5x macro lens was used to capture colour images. The body length was measured in the middle of the body following the elytral suture (the same for the pronotal and elytral length); width of the body means the maximum body width (usually the maximum span between lateral pronotal margins or span between outer margin of humeral callosities). Double slash (//) is used for the separation of data on different labels, square brackets ([]) for clarification of the text of the locality labels, and single slash (/) for the separation of lines of a text on a single label. Abbreviations of collections used in the text:

**MPCP** Mikuláš Plachetka collection, Prague, Czech Republic

**NMPC**National Museum, Prague, Czech Republic

## ﻿Taxonomy

### ﻿ Anthaxia (Merocratus) rydzii sp. nov.

Taxon classificationAnimaliaColeopteraBuprestidae

620DE6C9-5B1F-5A29-8C3E-85439753B719

https://zoobank.org/19C5B381-3B2C-4189-8D55-5BFBE4C57ADC

Figs 1–4 , 5–6 , 13 , 14

#### Type specimens.

***Holotype*** • ♂ (NMPC): Vietnam / Thanh Hóa / IV. 2024 / leg. local collector. ***Paratypes*** • 4♂ (MPCP): same data as holotype. All type specimens bear a red label with printed text: HOLOTYPE [PARATYPE respectively] / Anthaxia / (Merocratus) / rydzii sp. nov. / det M. Plachetka 2024.

#### Diagnosis.

Large, slender, green-black species. Dorsal body surface covered by sparse, white setae. Head retracted into prothorax, black-green, frons with wide but shallow, asymmetrically rounded depression. Eyes not projecting beyond outline of head. Pronotum green, with two large, indistinct, often fused, black or dark violet maculae. Antennae slightly overlapping mid-length of lateral pronotal margins when laid alongside. Pronotum widely depressed near posterior angles. Structure of pronotum consists of polygonal cells with central grains. In prescutellar part of pronotum cells smaller, with or without central grains, forming several feeble wrinkles. Scutellum cordiform, black or black-green. Elytra subparallel, weakly wedge-shaped, tapering from humeral callosities. Elytra 2.1 times as long as wide, green with black tinge and with indistinct black macula along suture. Elytra depressed from humeral callosities along lateral margin to apex. Transverse, basal depression deep, extending from anterior margin of humeral callosities to scutellum. Legs long, blue-green to violet, covered by sparse, white setae. Protibiae green, microdenticulated on inner margins with row of short brown setae. Mesotibiae slightly bent inward with short microdenticulation on apex of inner margins. Metatibiae bent inward with several distinct teeth near inner margin of apex, both outer and inner margins with row of brown setae. Tarsal claws and adhesive pads brown.

#### Description of holotype.

**Figures 1–4.**
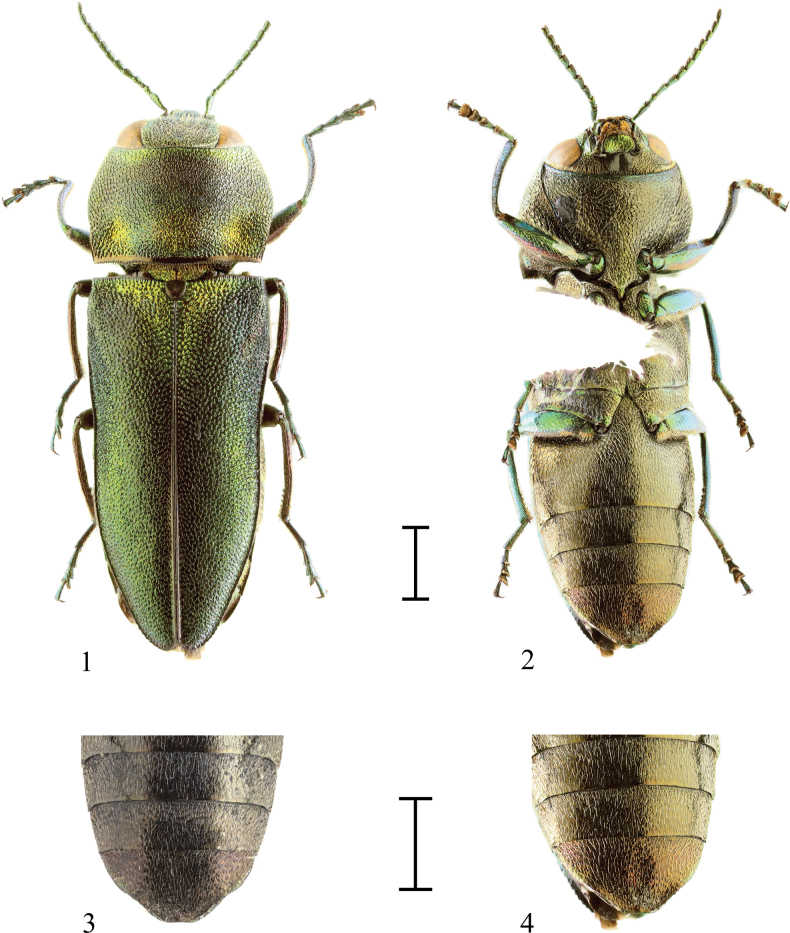
**1.**Anthaxia (Merocratus) rydzii sp. nov., holotype, NMPC (dorsal view); **2.**A. (M.) rydzii sp. nov., holotype (ventral view); **3.**A. (M.) rydzii sp. nov., holotype (anal sternite); **4.**A. (M.) rydzii sp. nov., holotype (anal sternite - different angle). Scale bars: 1.0 mm.

Holotype male (Figs 1–4, 14). Head retracted into prothorax, frons with wide but shallow, irregularly rounded depression. Frons green, partly with black tinge, vertex black. Sculpture of frons consists of irregularly rounded punctures with central grains, with white setae growing from central grains. Eyes large, reniform, not projecting beyond outline of head. Inner margin S-shaped. Antennae serrate, metallic green, rather long, overlapping mid-length of pronotum when laid alongside. Scape long, about 3.5 times as long as wide, claviform; pedicel barrel-shaped, approximately 2 times as long as wide; third antennomere rectangular, about 3 times as long as wide; antennomeres 4-10 obtusely trapezoidal; terminal antennomere oval, tip pointed.

Pronotum weakly convex, approximately 1.6 times as long as wide, with wide lateroposterior depressions, lateral margins irregularly rounded. Pronotum widest in the middle, anterior margin bisinuate, lobate in the middle, anterolateral angles acute. Posterior margin shallowly bisinuate. Depression in lateroposterior angles wide, not deep. Pronotum green with black tinge, in anterior part black tinge almost covers basic green colour. Black tinge forms two anteriorly fused asymmetrically triangular maculae. Sculpture of pronotum consists of irregular, polygonal cells, mostly with central grains with short white setae. In prescutellar part of pronotum horizontal border of cells forms short, feeble wrinkles (one wrinkle is formed by border of 3–6 cells), with shorter vertical borders. Scutellum cordiform, as long as wide, black, with very slight green tinge.

Elytra 2 times as long as wide, green with indistinct black macula around suture, partly with black tinge. Elytra subparallel, tapering from humeral callosities, depressed from humeral callosities to apex along margin, in second third depression widest, reaching half of elytral length. Elytral margins with fine microdenticulation, apices separately rounded. Sculpture of elytra consists of very fine wrinkles. Space between them often with small, deep, rounded punctures. Elytra with sparse, short, white pubescence. Deep basal, transverse depression above humeral callosities reaching scutellum. Green colour of elytral surface in this depression and in circumscutellar triangle lighter than in rest of elytra.

Ventral side black, partly with green or golden tinge, with lustrous reflection. Structure of pronotum consists of asymmetrically rounded cells with central grains with long white setae. Prosternum black, prosternal process with green tinge. Sternites black with green or golden tinge. All sternites slightly depressed near lateral margins, slightly truncate with irregularly S-shaped lateral margins. Anal sternite with irregularly circular depression in the middle and depression along apex, without notch. The shape of anal sternite significantly changes based on angle of view (Figs 3, 4).

Legs rather short, green to blue-green, covered with sparse, rather long, white setae. Protibiae slightly bent inward with pale brown setae along two-thirds of apical inner margins. Mesotibiae slightly bent inward, with pale brown setae only near apex of inner margin. Metatibiae bent inward with six distinct teeth near apex of inner margin, with pale brown setae on apical margins. Procoxae and mesocoxae green. Metacoxae black with green margins. Structure same as prosternum, outer lateroposterior angles acutely protruded, inner lateroposterior angle acute, forming small spine. Femora blue-green, metafemora and metatrochanters with white setae on lateral margins. Tarsi blue-green, adhesive pads and tarsal claws brown. Tarsal claw simple, only slightly enlarged near base.

Aedeagus (Fig. 13 – paratype, Fig. 14 – holotype) well sclerotized, stout. Parameres laterally, angulately widened, then narrowing, near apex with indistinct serration, with only few pale brown setae. Median lobe wide, with reticulate microsculpture, lateral margins as dark as parameres, central area and apex brighter. Apex tapering to rather wide, rounded tip.

#### Measurements.

Length: 7.0–7.4 mm (holotype 7.3 mm), width: 2.2–2.4 mm (holotype: 2.3 mm).

#### Variability.

**Figures 5–8.**
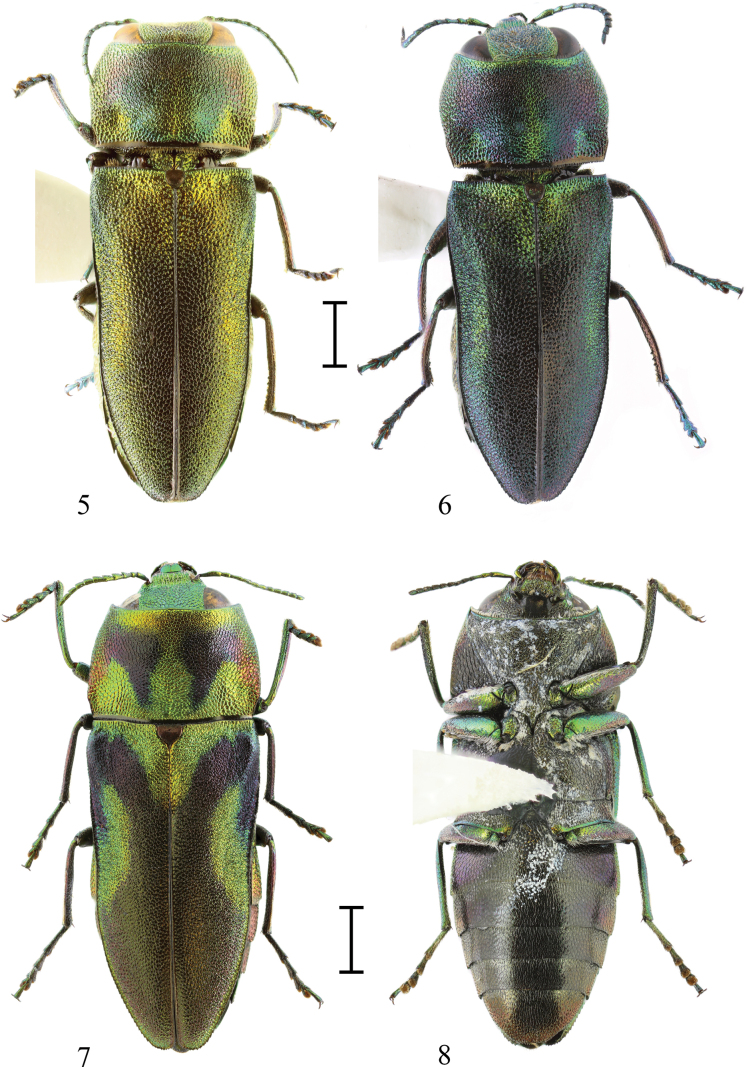
**5.**Anthaxia (Merocratus) rydzii sp. nov., paratype, MPCP—habitus variability (green colouration, black tinge); **6.**A. (M.) rydzii sp. nov., paratype, MPCP—habitus variability (violet tinge); **7.**A. (M.) barbieri Descarpentries, 1958, male, MPCP (dorsal view); **8.**A. (M.) barbieri Descarpentries, 1958, male (ventral view). Scale bars: 1.0 mm.

Among five known specimens, there are two paratypes very similar to the holotype, another specimen possesses almost completely green colouration, only with a black tinge (Fig. 5), and the last specimen has an elytral pattern similar to the holotype, but with a strong violet tinge on the pronotum (Fig. 6).

#### Sexual dimorphism.

Female unknown.

#### Bionomy.

Unknown.

#### Distribution.

Vietnam.

#### Etymology.

This species is dedicated to my friend, excellent photographer of (not only) living insects, co-author and colleague Daniel Rydzi from Králův Dvůr, Czech Republic.

#### Differential diagnosis.

**Figures 9–12.**
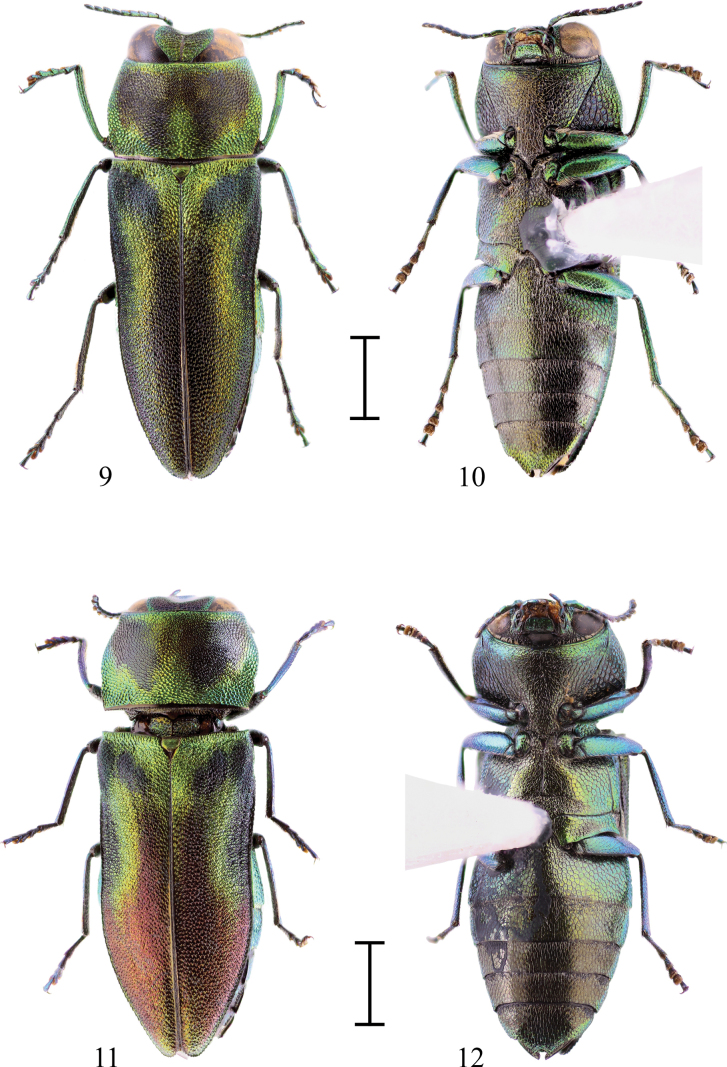
**9.**Anthaxia (Merocratus) angustata Bílý, 2019, male, MPCP (dorsal view); **10.**A. (M.) angustata Bílý, 2019, male (ventral view); **11.**A. (M.) angustata Bílý, 2019, female, MPCP (dorsal view); **12.**A. (M.) angustata Bílý, 2019, female (ventral view). Scale bars: 1.0 mm.

The closest species is Anthaxia (Merocratus) angustata Bílý, 2019 (Figs 9–12, 15) from northern Vietnam, which can be easily distinguished by its deeply depressed frons, anal sternite with a deep, rectangular notch, and the different shape of the aedeagus. Notch is a kerf replacing the tip of the apex of the anal sternite otherwise present in almost all subgenus Merocratus species. To illustrate the difference, an anal sternite of A. (M.) angustata (Figs 10, 12) can be compared to anal sternite of *A.rydzii* sp. nov. (Figs 3, 4) or to the anal sternite of another species similar to *A.rydzii* sp. nov.: A. (M.) barbieri Descarpentries, 1958, which also possesses an anal sternite without a notch (Fig. 8). However, A. (M.) barbieri can be distinguished by the straight metatibiae with only indistinct microdenticulation (metatibiae bent inward with several distinct teeth in A. (M.) rydzii sp. nov.), by more distinct elytral pattern and by slender, different aedeagus (Figs 7, 8, 16).

**Figures 13–16.**
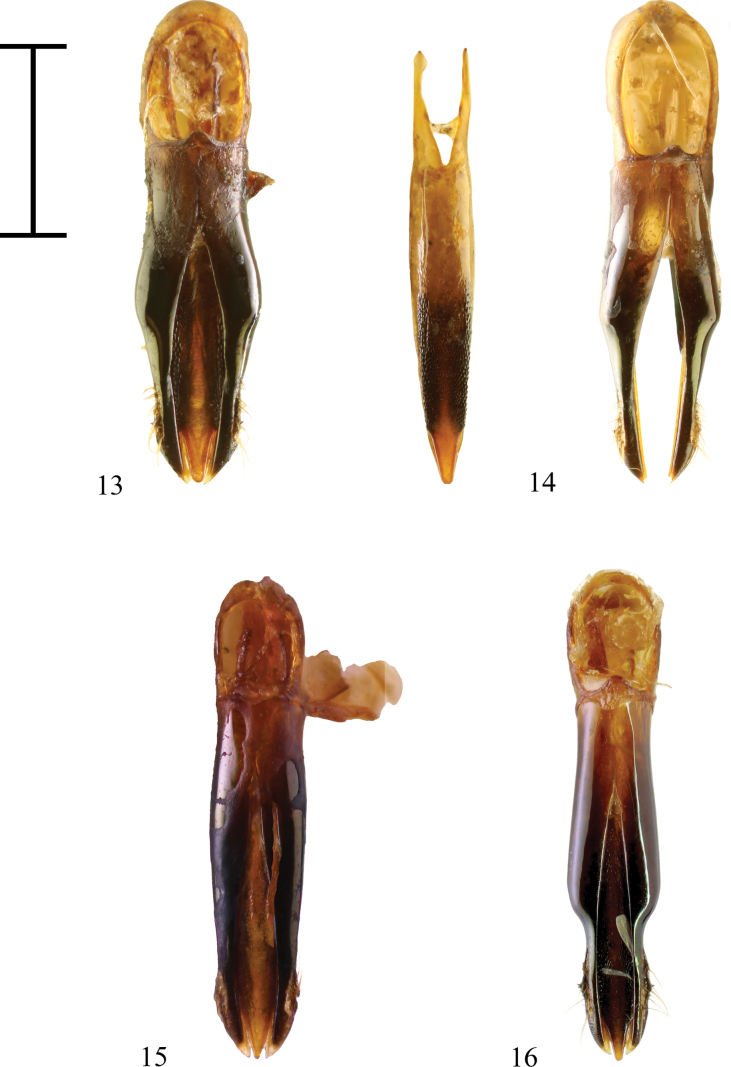
**13.**Anthaxia (Merocratus) rydzii sp. nov., paratype, MPCP (aedeagus); **14.**A. (M.) rydzii sp. nov., holotype, NMPC (parameres and median lobe); **15.**A. (M.) angustata Bílý, 2019, male, MPCP (aedeagus); **16.**A. (M.) barbieri Descarpentries, 1958, male, MPCP (aedeagus). Scale bar: 1.0 mm.

Anthaxia (M.) vietnamica Bílý, 1998 can be distinguished by its less bent metatibiae which lack apical teeth on the inner margin, by the different aedeagus and by the notched anal sternite.

Other species occurring in Vietnam—A. (M.) castanopsivora Bílý, 1998 and A. (M.) tamdaoensis Bílý, 1998—can be easily distinguished by the different elytra colour—all mentioned species have the elytra and postscutellar triangle either violet-black or bluish with green elytral maculae and by a notched anal sternite (Bílý 1990, 1998).

Anthaxia (M.) beesoniana Gebhardt, 1926 from Thailand possesses a similar anal sternite without a notch, but it can be distinguished by its different aedeagus, the black-violet basic colour of the elytra, and by the different structure of the pronotum.

### ﻿ Anthaxia (Merocratus) angustata

Taxon classificationAnimaliaColeopteraBuprestidae

Bílý, 2019

156ED405-DA64-51B5-BE16-11D788DE8B43

Figs 9–12 , 15

#### Type specimens examined.

Anthaxia (Merocratus) angustata Bílý, 2019: ***Holotype*** • ♂ (NMPC): N-Vietnam, Ninh Binh Pr[ovince]., Cuc Phuong NP [National Park], 270 m, 20°17.5727'N, 105°40.052'E, 22.–25.v.2015, leg. A. Weigel // HOLOTYPE / Anthaxia / (Merocratus) / angustata sp. nov. / Sv. Bílý det. [red label, printed].

#### Additional specimens examined.

Anthaxia (Merocratus) angustata Bílý, 2019: **Vietnam**: • 1♂, 1♀ (MPCP): VIETNAM V. 2023 / north Vietnam / Cao Bang / leg. local collector // Anthaxia (Merocratus) / angustata / Bílý 2019 / det. M. Plachetka 2024.

#### Discussion.

The female (Figs 11, 12) differs from the male (Figs 9, 10) by its stouter body and more convex elytra, which are parallel-sided in the anterior two-thirds, then tapering, apices separately rounded. Elytra shiny black with red tinge in posterior half, green elytral maculae same as in male. Ventral side black to dark blue-green with a shiny green stripe in the anterior margin of the prosternum and mesosternum, and with a narrow stripe along the lateral margins of the sternites. Anal sternite with a “Y” notch (in male notch widely rectangular).

### ﻿ Anthaxia (Merocratus) barbieri

Taxon classificationAnimaliaColeopteraBuprestidae

Descarpentries, 1958

2004D5B8-C0A3-56E9-A8B9-395C80BC2EA4

Figs 7 , 8 , 16

#### Specimens examined.

Anthaxia (Merocratus) barbieri Descarpentries, 1958: **Thailand**: • 1♂, 1♀ (MPCP): Thailand / Chiangmai / Chingdao hotel resort / 1. -15. 7. 2019 Ustinov V. // Anthaxia (Merocratus) / barbieri / Descarpentries / det. M. Plachetka 2023.

#### Discussion.

This species is similar to Anthaxia (Merocratus) rydzii sp. nov., but it can be distinguished by a more distinct elytral pattern and different aedeagus. Its aedeagus is slender and has a different shape (Fig. 16).

### ﻿Key to species of subgenus Merocratus Bílý, 1989 from Vietnam

**Table:** 

1	Frons flat or only slightly depressed, anal sternite without notch	**2**
–	Frons deeply depressed, anal sternite notched	**3**
2	Pronotum widest in posterior half, metatibiae without distinct teeth	***Anthaxiabarbieri* Descarpentries, 1958**
–	Pronotum widest in the middle, metatibiae with several distinct teeth	***Anthaxiarydzii* sp. nov.**
3	Dorsal colour rather bluish-violet, elytra with clearly bordered elytral maculae	**4**
–	Dorsal colour rather black-green, with unclearly bordered elytral maculae	**5**
4	Brighter, more shinier species, structure of pronotum in posterior angles consist of prolonged cells without central grains, anal sternite with parallel-sided notch	***Anthaxiatamdaoensis* Bílý, 1998**
–	Darker species, structure of pronotum in posterior angles consist of smaller, irregular polygonal cells with central grains, anal sternite with subparallel-sided notch	***Anthaxiacastanopsivora* Bílý, 1998**
5	Black-green species with green scutellum, frons green with black tinge	***Anthaxiaangustata* Bílý, 2019**
–	Black-green species with black scutellum, frons green with blue tinge	***Anthaxiavietnamica* Bílý, 1998**

## Supplementary Material

XML Treatment for Anthaxia (Merocratus) rydzii

XML Treatment for Anthaxia (Merocratus) angustata

XML Treatment for Anthaxia (Merocratus) barbieri

## ﻿Additional information

### Use of AI

No use of AI was reported.

### Author ORCIDs

Mikuláš Plachetka https://orcid.org/0009-0004-1246-1186

### Data availability

All of the data that support the findings of this study are available in the main text.
